# Case report: Case analysis of multiple sclerosis with preclinical systemic lupus erythematosus presenting as rare bilateral horizontal gaze palsy

**DOI:** 10.3389/fimmu.2024.1453264

**Published:** 2024-09-05

**Authors:** Li Huan, Yu Xiangming

**Affiliations:** Department of Neurology, 970th Hospital of PLA Joint Logistic Support Force, Yantai, China

**Keywords:** multiple sclerosis, systemic lupus erythematosus, undifferentiated connective tissue disease, diagnostic criteria, binocular dyskinesia

## Abstract

We present an analysis of a case initially manifesting as bilateral horizontal gaze palsy, eventually diagnosed as multiple sclerosis (MS) with preclinical systemic lupus erythematosus (p-SLE). The patient, a 25-year-old male, exhibited restricted movement in both eyes. Cranial MRI revealed multiple demyelinating lesions; serum analyses indicated elevated levels of antinuclear antibodies (ANA), anti-Sm antibodies, and anti-nRNP antibodies. Oligoclonal bands were identified in the cerebrospinal fluid. Neurophysiological assessments demonstrated damage to the optic, auditory, and facial nerves. Given the clinical presentation, laboratory findings, and the progression of the disease, the final diagnosis was confirmed as MS associated with p-SLE. The onset of MS with oculomotor disturbances is rare and may be easily confused with neuropsychiatric systemic lupus erythematosus (NPSLE). Furthermore, the differentiation of p-SLE from undifferentiated connective tissue disease (UCTD) in the early stages presents significant challenges. Early identification of risk factors and close monitoring of disease activity is crucial for an accurate diagnosis.

Multiple Sclerosis (MS) is a chronic, immune-mediated disease that affects the central nervous system, typically developing in adults between the ages of 20 and 40; it is more prevalent in women than in men. Symptoms of initial MS episodes can be highly diverse, ranging from asymptomatic to severe disabilities, such as vision loss, diplopia, eye movement abnormalities (e.g., horizontal or vertical gaze palsy), sensory disturbances, and motor or autonomic dysfunctions (1). Ocular dyskinesia is a common manifestation of MS, affecting up to 75% of patients with abnormal eye movements at some point during the disease course (2). However, among all symptoms, bilateral horizontal gaze palsy is exceedingly rare (3).

Systemic Lupus Erythematosus (SLE) is an autoimmune disease with multi-system involvement that can affect the central and peripheral nervous systems, leading to a number of neuropsychiatric symptoms known as NPSLE. When SLE affects the peripheral nervous system, damage to multiple groups of cranial nerves is one of its clinical manifestations. In patients with SLE, multiple groups of cranial nerves may be damaged simultaneously or sequentially, commonly involving the optic nerve, auditory nerve, facial nerve, motoneuron, and spreading nerve (4), resulting in clinical manifestations such as decreased visual acuity, facial numbness, and impaired eye movement. It has been suggested that the presence of cranial nerve damage may correlate with the disease activity of SLE (5). Consequently, differentiating between Multiple Sclerosis (MS) and NPSLE based solely on clinical symptoms was challenging before imaging and antibody tests had been conducted in this patient.

This article reports a rare case of multiple sclerosis with preclinical systemic lupus erythematosus(p-SLE) starting with bilateral horizontal gaze palsy, and analyzes the patient’s diagnostic thinking in the early stages of the disease, aiming to improve clinicians’ understanding of this disease.

## Clinical information

A 25-year-old male was admitted with persistent limited movement of both eyeballs for one day. The day before admission, he noticed limited eyeball movement upon waking, which did not improve with rest. Absent of fever, headache, mental abnormalities, speech difficulties, or limb dyskinesia, he sought medical consultation and was admitted to investigate ocular dyskinesia. The patient’s medical and personal histories were unremarkable. During the physical examination, he was alert, articulate, and demonstrated normal cognitive functions. Visual acuity was normal; pupils were symmetrical, round, approximately 3.0 mm in diameter, with responsive light reflexes. Eyeball movements were completely restricted horizontally. No nystagmus or diplopia was present; other cranial nerve exams were normal. Limb muscle strength was grade 5 with normal muscle tone; no ataxia was noted. Facial hyperalgesia was present; limb sensation was unimpaired. All reflexes were active; Babinski’s and Chaddock’s signs were negative. Neck tenderness, Kirschner’s, and Bucher’s signs were absent. Additionally, the patient did not exhibit any rash, butterfly-shaped erythema of the cheeks, or joint pain.

**Figure 1:**
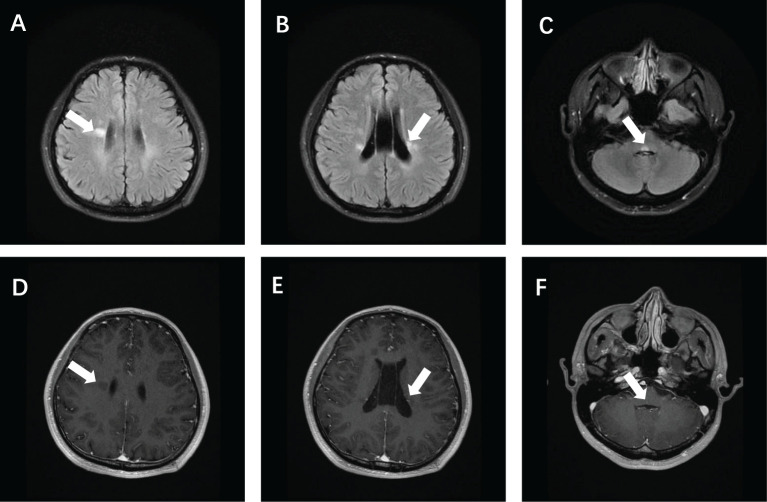
Brain magnetic resonance imaging of the patient showed T2 Flair hyperintensity in the bilateral periventricular **(A, B)** and brain stem **(C)**, and enhanced showed partial lesion enhancement **(E)** and partial lesion non-enhancement **(D, F)**.

After admission, tests showed: an antinuclear antibody spectrum with anti-ANA at 1:640, positive anti-Sm, strongly positive anti-nRNP; normal complete blood count, creatine kinase at 223 U/L, cholinesterase at 13660 U/L; normal thyroid function, no infectious diseases; negative tests for new coronavirus and respiratory pathogens; and normal blood sedimentation, rheumatologic markers, immunoglobulins, and complements. Additionally, the patient tested negative for antiphospholipid antibodies. Cranial MRI revealed multiple high signals on T2 Flair in bilateral radiocoronal areas and brainstem, with enhanced lesions noted (**Figure 1**). Cranial MRA and MRV showed a slender right sigmoid and transverse sinus. Cervical spine MRI showed C5/6, C6/7 disc herniations, suggestive of a narrowed right vertebral artery. Additional diagnostics, including chest CT, cardiac ultrasound, and abdominal ultrasound, revealed no abnormalities.

Visual evoked potentials showed prolonged P100 latency on the right; brainstem auditory evoked potentials indicated prolonged right wave III latency, with other metrics normal. Electromyography revealed demyelinating changes in the bifacial nerves. These comprehensive diagnostic findings led to a preliminary diagnosis of multiple sclerosis. Following admission, high-dose hormone shock therapy began with 1000 mg of methylprednisolone for 3 days, reduced to 60 mg of oral prednisone, supplemented by Chinese acupuncture. The patient’s ocular dyskinesia resolved completely, and he was discharged for regular follow-up.

## Discussion

MS is an inflammatory demyelinating disease affecting the central nervous system, with pathogenesis closely associated with autoimmune disorders. Diagnosis of MS typically occurs when patients present with characteristic symptoms, yet the clinical manifestations vary widely depending on the lesion location within the central nervous system. This variability can lead to underdiagnosis or misdiagnosis, particularly due to insufficient clinical evidence in the early stages of the disease (6). In our patient, the initial symptom was a binocular horizontal movement disorder. Visual evoked potentials, auditory evoked potentials, and electromyography indicated injuries to multiple cranial nerve groups. Commonly, MS presents with sensory problems, visual disturbances, muscle weakness, and oculomotor deficits. Specifically, MS-related oculomotor deficits typically manifest as internuclear ophthalmoplegia, nystagmus, or nuclear ophthalmoplegia, rather than binocular horizontal movement deficits (2). Therefore, when patients present with a broader spectrum of clinical symptoms beyond the unusual initial symptom of binocular horizontal dyskinesia, differentiation from other potential autoimmune disorders, such as neuropsychiatric systemic lupus erythematosus (NPSLE),is necessary (1–3).

Imaging studies have revealed differences between MS and NPSLE: MS lesions typically favor periventricular and proximal cortical areas, while lesions associated with SLE tend to be more extensive, affecting both deep cerebral white and gray matter. MS lesions are usually well-defined and oval or striated, while those in SLE often exhibit fuzzy borders and diverse morphologies. On T2-weighted imaging, MS lesions typically show a high signal, and on T1-weighted imaging, they may display a low or equal signal. In contrast, SLE lesions often show high signals on both T1- and T2-weighted imaging. Furthermore, MS lesions may exhibit no or slight enhancement on enhancement scans, while those in SLE can display more pronounced enhancement. Over time, MS lesions can present as new active lesions or exhibit enlargement of existing lesions, whereas lesions in SLE tend to be more stable or progress slowly (7). Studies utilizing radiomics to analyze patients with MS and NPSLE have demonstrated that calculating a radiomics index for each lesion enables the identification of MS and NPSLE at an early clinical stage and improves diagnosis by comparing the distance between the RIL value and the threshold (8). Therefore, the imaging features of this patient, exhibiting both spatial and temporal multiplicity, were consistent with MS rather than NPSLE. Ultimately, combined with the cerebrospinal fluid examination results, the patient was definitively diagnosed with MS according to McDonald’s diagnostic criteria.

Elevated titers of anti-ANA, anti-nRNP, and anti-Sm antibodies in our patient strongly suggest a risk of SLE; however, a review of the patient’s medical history revealed no clinical signs other than oral ulcers. Research conducted in Korean hospitals has shown that 14.4% of patients tested for ANA were positive, yet less than 1% of these cases resulted in diagnoses of ANA-associated rheumatic diseases, underscoring the need for cautious interpretation of ANA tests (9). In patients with MS, there is a significant prevalence of ANA, which reflects underlying immune system abnormalities. Research suggests that MS patients often have higher ANA levels, suggesting a complex autoimmune profile. These findings underscore the critical importance of understanding the role of ANAs in both healthy individuals and those with autoimmune diseases such as MS, to ensure accurate diagnosis and management (10). SLE is an autoimmune disease characterized by the presence of multiple autoantibodies. The presence of these autoantibodies can be observed in the preclinical phase of SLE. These autoantibodies appear in a specific sequence: initially, ANA, antiphospholipid, anti-Ro (SS-A), and anti-La (SS-B) antibodies emerge, followed by anti-double-stranded DNA (dsDNA) antibodies a few months prior to clinical diagnosis. Levels of anti-Sm and anti-nRNP antibodies exponentially increase in the year preceding the diagnosis, reaching their peak before the disease is formally recognized. The presence of these autoantibodies not only predicts the onset of SLE but also correlates with its activity and severity (11).

Three major SLE classification criteria exist: the ACR-1997, SLICC-2012, and EULAR/ACR-2019 criteria. The ACR-1997 criteria demonstrate high specificity but low sensitivity, while the SLICC-2012 and EULAR/ACR-2019 criteria achieve increased sensitivity through varied scoring systems, facilitating the early recognition of SLE. Specifically, the EULAR/ACR-2019 criteria emphasize early diagnosis and broad applicability across various genders and races (12). Furthermore, SLERPI, a novel machine-learning-based tool, exhibits high sensitivity but low specificity, and has not yet gained wide acceptance (13). In certain instances, SLERPI may detect SLE earlier than the EULAR/ACR-2019 criteria. Based on this patient’s clinical presentation and abnormal antibody results, only the SLERPI scale was able to directly diagnose SLE among the four scales; the others did not provide a definitive diagnosis. Although SLERPI has not yet been accepted by major international organizations, a provisional diagnosis of this patient with SLE appears more justified.

Differentiating p-SLE from UCTD in clinical practice presents significant challenges due to overlapping clinical presentations. Moreover, UCTD can progress to SLE. A long-term follow-up study showed that approximately 11.3% of patients with UCTD progressed to definite CTD after a median of 11 years, with a subset advancing to SLE (14). Another study demonstrated that lower age, plasma membrane inflammation, and the presence of anti-dsDNA, anti-Sm, anti-Ro, and anti-cardiolipin antibodies were predictive of UCTD progressing to SLE (15). UCTD typically presents with milder symptoms, while p-SLE manifests more severe clinical symptoms, including skin manifestations, arthritis, pleurisy, and pericarditis (6). ANA may be positive in both conditions, yet patients with p-SLE often present additional antibodies such as anti-dsDNA and anti-Sm, exhibiting more definitive signs and symptoms over a shorter period (16). Over time, some patients with UCTD may progress to definitive SLE or other CTDs, while others remain stable without clear classification into any specific CTD. The duration from the onset of signs or symptoms to diagnosis represents an important variable. A diagnosis of UCTD is more tenable if the patient shows no new clinical and laboratory manifestations for three years and lacks specific serologic features of CTDs. Clinician judgment is crucial for differentiating UCTD from p-SLE, necessitating a synthesis of clinical presentation, laboratory results, and disease progression (15). The findings from the aforementioned studies substantiate the diagnosis of p-SLE as reasonable for this patient.

In summary, the preclinical phase of autoimmune diseases is typically prolonged, often lasting many years, and characterized by asymptomatic or mild symptoms. This suggests that autoimmune diseases develop through a gradual evolutionary process driven by interactions between genetic and environmental factors. In MS and p-SLE, the diagnostic challenge involves recognizing early, potentially nonspecific signs and symptoms, and correlating them with the eventual clinical presentation. The progression of UCTD to SLE involves a complex interplay of clinical, laboratory, and therapeutic factors. Early identification of risk factors and meticulous monitoring of disease activity are essential to mitigating or slowing disease progression.

## Data Availability

The datasets presented in this article are not readily available because of ethical and privacy restrictions. Requests to access the datasets should be directed to the corresponding author.
